# Oncogenic function and prognostic significance of protein tyrosine phosphatase PRL-1 in hepatocellular carcinoma

**DOI:** 10.18632/oncotarget.1986

**Published:** 2014-05-19

**Authors:** Shaowen Jin, Kaimei Wang, Kang Xu, Junyao Xu, Jian Sun, Zhonghua Chu, Dechen Lin, Phillip H. Koeffler, Jie Wang, Dong Yin

**Affiliations:** ^1^ Department of Hepatobiliary Surgery, Sun Yat-Sen Memorial Hospital, Sun Yat-Sen University, Guangzhou, China; ^2^ Guangdong Provincial Key Laboratory of Malignant Tumor Epigenetics and Gene Regulation, Medical Research Center, Sun Yat-Sen Memorial Hospital, Sun Yat-Sen University, Guangzhou, China; ^3^ Department of Pediatrics, Sun Yat-Sen Memorial Hospital, Sun Yat-Sen University, Guangzhou, China; ^4^ Department of Gastrointestinal Surgery, Sun Yat-Sen Memorial Hospital, Sun Yat-Sen University, Guangzhou, China; ^5^ Division of Hematology/Oncology, Cedars-Sinai Medical Center, University of California-Los Angeles (UCLA) School of Medicine

**Keywords:** Hepatocellular carcinoma, PRL-1, SNP-Chip, Tumor metastasis, Prognostic marker

## Abstract

Our SNP-Chip data demonstrated 7/60 (12%) hepatocellular carcinoma (HCC) patients had PRL-1 copy number amplification. However, its biological functions and signaling pathways in HCC are deficient. Here, we investigated its oncogenic function and prognostic significance in HCC. PRL-1 protein levels were examined in 167 HCC samples by immunohistochemisty (IHC). The relationship of PRL-1 expression and clinicopathological features was assessed by correlation, Kaplan-Meier and Cox regression analyses. The oncogenic function of PRL-1 in HCC cells and its underlying mechanism were investigated by ectopic overexpression and knockdown model. PRL-1 levels in primary HCC and metastatic intravascular cancer thrombus were also determined by IHC. PRL-1 levels were frequently elevated in HCC tissues (81%), and elevated expression of PRL-1 was significantly associated with more aggressive phenotype and poorer prognosis in HCC patients (*p*<0.05). Ectopic overexpression of PRL-1 markedly enhanced HCC cells migration and invasion. Furthermore, the oncogenic functions of PRL-1 were mediated by PI3K/AKT/GSK3β signaling pathway through inhibiting E-cadherin expression. Finally, PRL-1 protein levels in metastatic cancer thrombus were higher than that in primary HCC tissues (*p*<0.05). These data highlight the oncogenic function of PRL-1 in HCC invasion and metastasis implicating PRL-1 as a potential prognostic marker as well as therapeutic target in HCC.

## INTRODUCTION

Hepatocellular carcinoma (HCC) is the fifth most common cancer worldwide and the third most common cause of cancer mortality [1,2]. More than 75% of HCC patients develop recurrence and/or metastasis within 5 years after surgery becoming the main cause of death for these individuals [3]. Reversible tyrosine phosphorylation is critical for regulating the signaling pathways involved in tumor cell adhesion, invasion, and metastasis, It is governed by the balance between protein tyrosine kinases (PTKs) and protein-tyrosine phosphatases (PTPs) [4]. Compared with the large family of PTKs, PTPs superfamily has been less intensively studied. The phosphatase of regenerating liver (PRL) subgroup of PTPs, a unique class of prenylated phosphatases, had received much attention since PRL-3 was found to be the only gene consistently overexpressed in all 18 metastatic colorectal cancers [5].

Among the PRL family, PRL-3 is the most thoroughly investigated and its upregualtion is associated with progression and metastasis of several types of human cancer, including colorectal, breast, and gastric tumors [6-8]. PRL-3 shows promise as a potential biomarker of cancer prognosis as well as therapeutic target of cancers [9]. In contrast, the role in cancer of PRL-1 and PRL-2, the other two members of PRLs family, is less well studied. PRL-1 phosphatase (also known as PTP4A1) was originally identified as an immediate early gene in regenerating liver after partial hepatectomy and mitogen-stimulated cells [10,11]. Subsequent studies revealed that PRL-1 expression was elevated in several tumor cell lines, and ectopic overexpression of PRL-1 enhanced tumor cell proliferation [12], clonogenic growth in soft agar [11], as well as migration and invasion [13,14]. Importantly, the expression of PRL-1 enhanced metastatic potential of tumors. Zeng et al [13] demonstated that following the injection of Myc-PRL-1-expressing CHO cells into the tail vein of mice, induced lung metastases occurred, whereas control cells did not form lung tumors. A similar experiment [6] found that both the number and volume of hepatic metastatic foci decreased significantly in the mice injected with PRL-1-small interfering RNA-treated DLD-1 cells as compared to control cells. These results suggested an oncogenic role for PRL-1 in cancer progression and metastasis.

Functionally, PRL-1 phosphatase has been linked to several pathways [15], including the regulation of diverse focal adhesion components (e.g. p130Cas, Src, FAK) [16,17], Rho GTPases family [14], ERK1/2 [18] as well as MMPs [19]. However, the fuction of PRL-1 in cancer cells including liver cancer, is poorly understood, even though PRL-1 phosphatase was first discovered in regenerating liver tissue. In this report, we show that (i) PRL-1 is very frequently amplified and overexpressed in hepatocellular carcinoma; (ii) PRL-1 represses E-cadherin expression associated with enhancing hepatoma cells migration and invasion in concert with activating the PI3K/AKT signaling pathway; (iii) Elevated level of PRL-1 is associated with intravascular metastasis and an independent prognostic factor for poor survival for patients with HCC.

## RESULTS

### Copy number amplification and higher protein levels of PRL-1 are frequently detected in HCC tissues

**Figure1:**
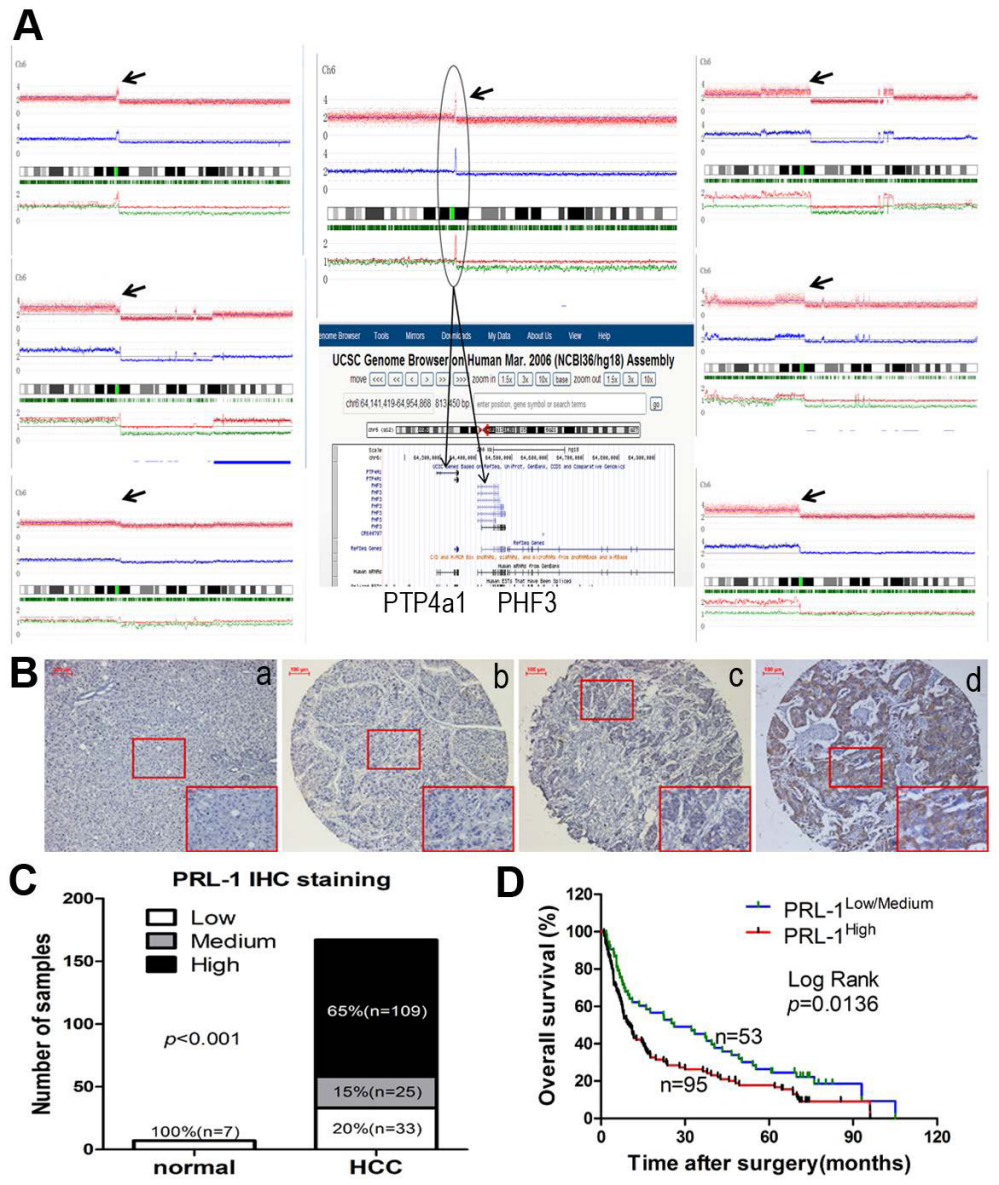
Copy number analysis, expression levels and prognostic value of PRL-1 in patients with HCC A, 7 cases had amplification of Chr6:q12 (black arrow). B. Different immunohistochemical intensity showing that (a) represents low expression in normal liver tissue and (b-d) represent low, medium, and high expression in HCC tissues, respectively. Magnification: x200. C, Expression status of PRL-1 was determined in 7 normal liver and 167 HCC samples by IHC. PRL-1 was significantly overexpressed in HCC tissues (Kruskal Wallis Test, *p*<0.001). D, Kaplan–Meier survival analysis of PRL-1 expression in HCC patients (*p*=0.0136).

DNA copy number analysis of HCC samples showed that 7/60 (12%) samples had amplification of Chr6:q12, in two cases which had a narrow region of Chr6:q12 with high DNA copy number. The common minimum amplified region (CMAR) of chr6 (q12) encompassed only the PRL-1 and PHF3 genes (Figure 1A). Moreover, PRL-1 is expressed as an immediate-early gene in regenerating liver [11], prompting us to focus further on this gene. Protein expression level of PRL-1 was examined in 167 HCC specimens and 7 normal liver samples by IHC. The immunohistochemical staining intensity was divided into low, medium and high groups (Figure 1B). Compared with normal liver tissues, levels of PRL-1 were significantly higher in HCC samples. 65% (109/167) of HCC cases had high staining, 15% (25/167) with medium staining and 20% (33/167) low staining (Figure 1C). In contrast, all 7 non-neoplastic liver tissues displayed low immunohistochemical staining. Integrative analysis of both our SNP-Chip and protein expression data strongly suggests that PRL-1 may function as an oncogene in HCC.

### High-level protein expression of PRL-1 in HCC is associated with an aggressive and/or poor prognostic phenotype

The clinicopathological and prognostic significance of PRL-1 protein levels in HCC patients were examined using correlation, Kaplan-Meier and Cox regression analyses. High PRL-1 protein level was significantly correlated with a more aggressive tumor phenotype as measured by TNM stage as well as cancer thrombus (*p*<0.05, Table1). The median overall survival (OS) of high-level PRL-1 expression group (10.07 months) was much shorter than the OS of the low/medium-level group (26.07 months) (*p*=0.0136, Figure 1D). Univariate Cox regression analysis also indicated those patients whose HCC had high expression of PRL-1 had shorter OS (*p*=0.012, Table 2). Furthermore, multivariate Cox regression analysis revealed that high-level expression of PRL-1 is an independent prognostic factor for poor OS of patients with HCC (*p*=0.017, Table 2).

**Table:** Table 1. Correlation of PRL-1 Expression Levels with Clinicopathological Status in HCC Patients

Variables^a^	PRL-1		P-value^b^
	low/medium	high	
Age(years)			0.100
<45	15	42	
≥45	43	67	
Gender			0.885
male	49	93	
female	9	16	
AFP(ug/L)			0.122
<400	37	56	
≥400	20	51	
HBV infection			0.886
(−)	7	14	
(+)	51	95	
Tumor size(cm)			0.399
>5	17	39	
≥5	41	70	
cirrhosis			0.013*
(−)	20	19	
(+)	38	90	
TNM stage			0.035*
I	33	43	
II/III	25	65	
Histological grade			0.534
I	21	35	
II	24	38	
III	12	30	
cancer thrombus			0.048*
(−)	38	54	
(+)	20	55	

a Cases with missing data were not included for analysis

b p-value by Chi-square test

AFP, alpha-fetoprotein; HBV, hepatitis B virus; TNM, tumour-node-metastasis

* p<0.05

**Table2:** Univariate and multivariate analysis of factors associated with overall survival of patients with hepatocellular carcinoma

Vavirable	cases number	HR(95%CI)	p value
Univariate analysis			
Age(years)			0.049
<45 vs ≥45	50/90	0.690(0.477-0.999)	
Gender			0.014
Male vs Female	118/22	1.814(1.128-2.915)	
AFP(ug/L)			0.040
<400 vs ≥400	77/63	1.454(1.018-2.078)	
HBV infection			0.772
(−) vs (+)	19/121	1.098(0.657-1.834)	
Tumor size(cm)			0.003
<5 vs ≥5	44/96	1.807(1.221-2.675)	
Cirrhosis			0.211
(−) vs (+)	36/104	1.311(0.858-2.003)	
TNM stage			0.000
I vs II/III	65/75	2.046(1.426-2.937)	
Histological grade			0.004
I/II vs III	102/38	1.798(1.203-2.687)	
Cancer thrombus			0.000
(−) vs (+)	71/69	2.038(1.420-2.925)	
PRL-1(expression)			0.012
Low/midum vs High	51/89	1.629(1.116-2.377)	
Multivariate analysis			
Gender			0.004
Male vs Female	118/22	2.026(1.249-3.286)	
PRL-1(expression)			0.017
Low/Medium vs High	51/89	1.584(1.084-2.316)	
TNM stage			0.000
I vs II/III	65/75	2.318(1.478-3.091)	

HRs(95% CI) and p values were calculated using univariate or multivariate Cox proportional hazard regression in 140 cases

AFP, alpha-fetoprotein; HBV, hepatitis B virus; TNM, tumour-node-metastasis

### Exogenous overexpression of PRL-1 enhanced migration and invasion of hepatoma cells *in vitro*

**Figure2:**
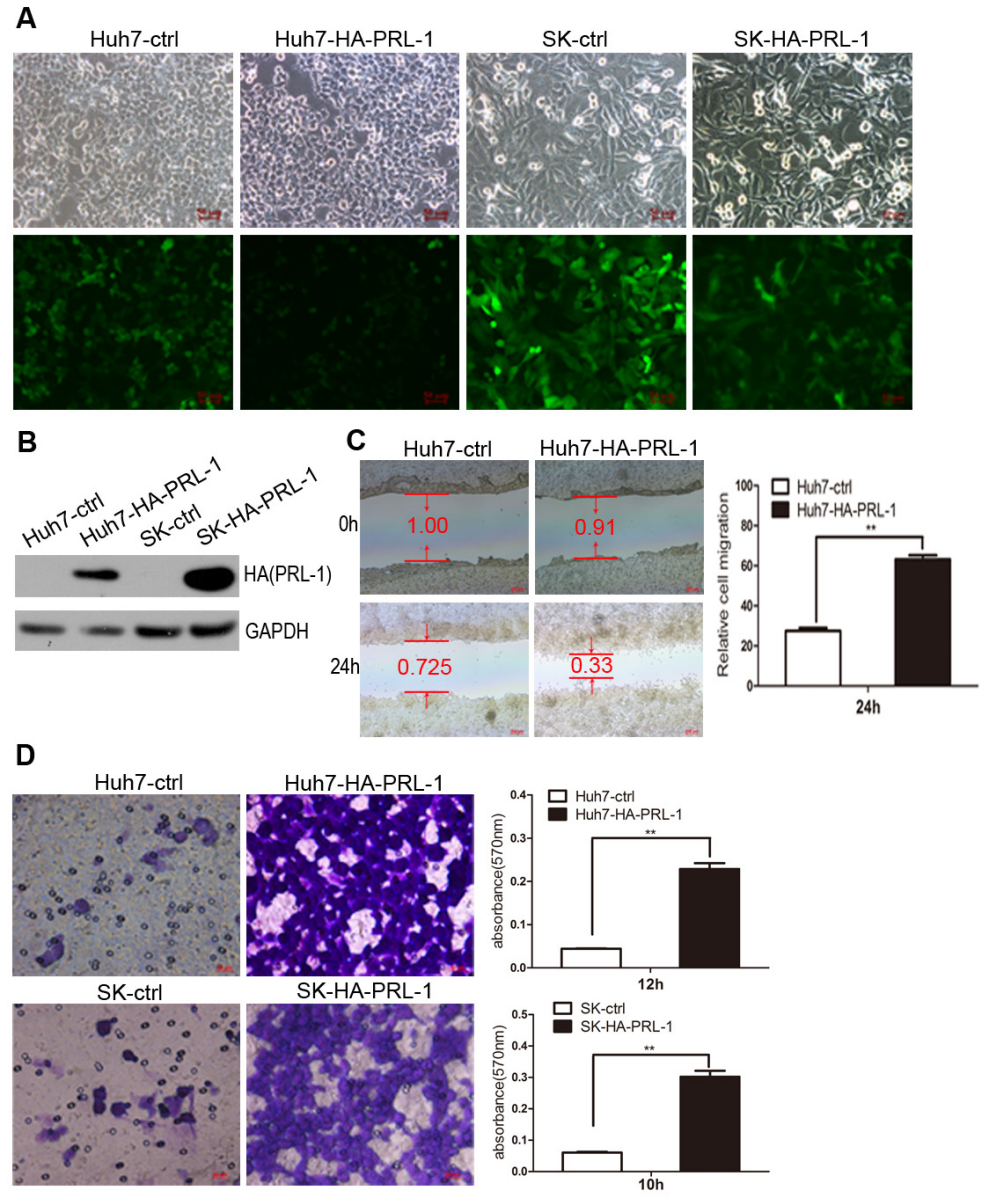
Exogenous expression of PRL-1 promotes hepatoma cell migration and invasion A and B, PRL-1 and empty vector stably-transfected HCC cell lines were successfully constructed by pMSCV-PIG retrovirus infection. C, Ectopic overexpression of PRL-1 remarkbely enhanced Huh7 cells migration. D, PRL-1 stably-transfected Huh7 and SK-hep1 cells showed significantly higher invasive capacity compared with their respective control cells.

The above observations prompted us to explore the potential biological function of PRL-1 in HCC progression. HCC cell lines were stably transfected with pMSCV-HA-PRL-1 expression vector (Figure 2A,B). Those cells with forced expression of PRL-1had greaten ability to migrate compared to control (ctrl) cells as measured by both a wound-healing assay and a Matrigel invasion assays using Huh7-HA-PRL-1 and SK-hep1-HA-PRL-1 cells versus vector contro cells (p<0.01, Figure 2C,D).

### PRL-1 inhibits E-cadherin expression in HCC cell lines and has inverse correlation with E-cadherin expression in HCC tissues

**Figure3:**
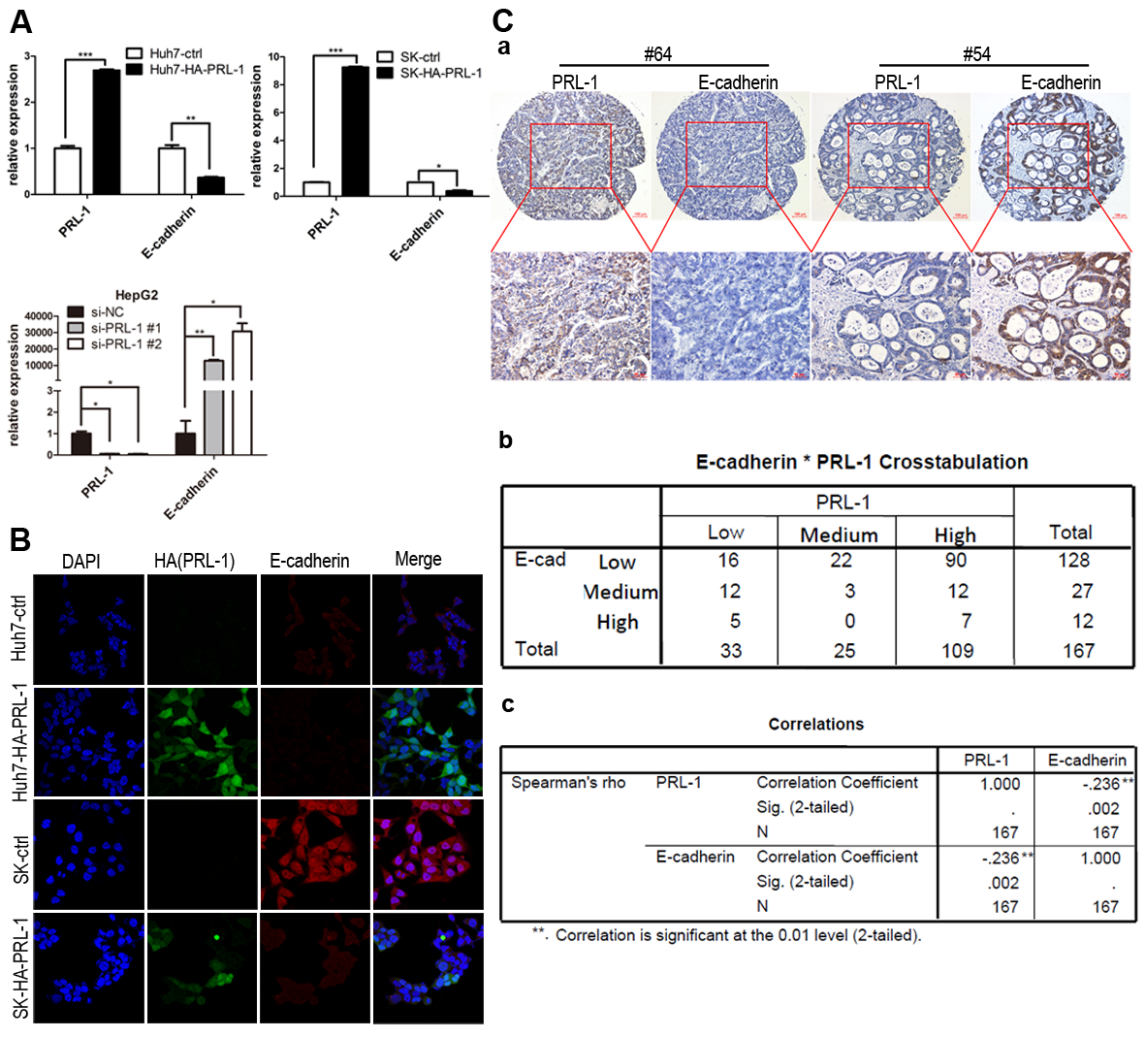
PRL-1 represses the expression of E-cadherin at both the mRNA and protein levels and negatively correlates with E-cadherin expression in clinical HCC samples A, Reintroduction of PRL-1 markedly inhibited E-cadherin expression at mRNA level in Huh7 and SK-hep1 cell lines; the mRNA level of E-cadherin was elevated substantially after Knockdown PRL-1 expression in HepG2 cell line by small interfering RNA (siRNA). B, Immunofluorescence was used to compare the expression levels of E-cadherin between PRL-1 stably-transfected cell lines and their respective control cell lines. PRL-1 (green signal) significantly suppressed E-cadherin (red signal) expression in Huh7 and SK-hep cell lines. Magnification: x400. C, PRL-1 expression inversely correlates with E-cadherin expression in HCC tissues as measured by IHC. (a), two examples of association of PRL-1 with E-cadherin expression. The upper panels, low magnification (x100); the lower panels, high magnification (x200). (b), Correlation analysis reveals that PRL-1 is significantly asscociated with E-cadherin expression (*p*=0.002)

**Figure4:**
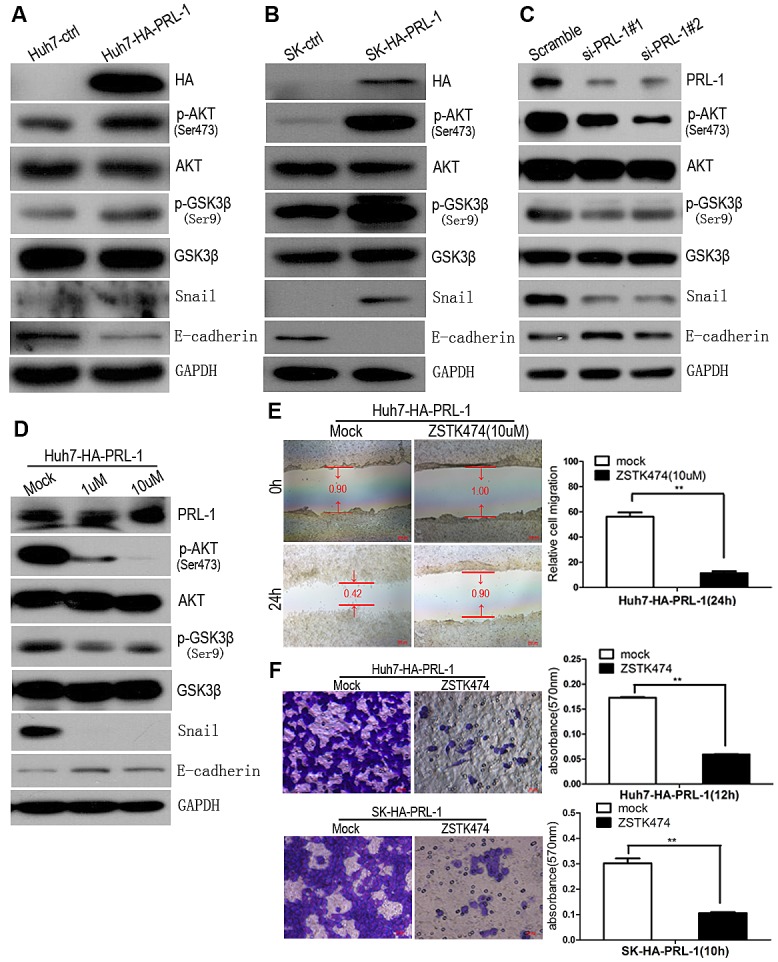
PRL-1 suppresses E-cadherin expression through PI3K/AKT signaling pathway and PI3K/AKT signals mediate PRL-1-enhanced migration and invasion in HCC cells A and B, Exogenous overexpression of PRL-1 enhanced phosphorylation of AKT at Ser^474^, inactivated GSK-3β at Ser^9^, upregulated expression of Snail transcriptional factor, and downregulated E-cadherin expression in Huh7 and SK-hep1 cell lines by Western blot assay. C, Knockdown PRL-1 expression by small interfering RNA approach inhibited p-AKT (Ser^474^), activated GSK-3β(Ser^9^), downregulated Snail expression, and reversed E-cadherin expression. D, Treatment of Huh7-HA-PRL-1 cells with PI3K inhibitor, ZSTK474, suppressed phosphorylation of AKT at Ser^474^, stabilized GSK-3β, reduced Snail expression, and restored the experssion of E-cadherin. ZSTK474 (10uM) markedly suppressed PRL-1-induced migration in SK-hep1 cells (E) and invasion in Huh7 and SK-hep1 cells (F). Scale bar: 20 um.

Endothelial-mesenchymal transition (EMT) provides cancer cells with invasive and metastatic properties [20]. We investigated whether PRL-1 may be involved in regulation of EMT in HCC. Expression of E-cadherin was down-regulated after ectopic overexpression of PRL-1 in Huh7 and SK-hep1 cells (Figure 3A). In contrast, silencing PRL-1 expression by small interfering RNA (siRNA) caused a marked increase of E-cadherin mRNA levels in the HepG2 cell line (p<0.05, Figure 3A). Congruent with the RNA data, western blots showed that E-cadherin protein levels decreased after reintroduction of PRL-1 in Huh7 and SK-hep1 cell lines, and knockdown of PRL-1 expression caused a marked enhancement of E-cadherin protein level in Huh7 cells (Figure 4A,B,C). Furthermore, immunofluorescence assay confirmed that forced expression of PRL-1 down-regulated E-cadherin protein level in Huh7 and SK-hep1 HCC cell lines (Figure 3B). Taken together, these results indicate that PRL-1 regulated E-cadherin expression in HCC cells at both the mRNA and protein levels.

The relationship between PRL-1 and E-cadherin expression was further evaluated by IHC in 167 clinical HCC samples. A total of 83% (90/109) of HCC samples with high PRL-1 expression level displayed low E-cadherin expression. Likewise, 44% (17/39) of cases with high level of E-cadherin had low expression level of PRL-1. Correlation analysis found that a significant negative correlation existed between expression of PRL-1 and E-cadherin in HCC [*p*=0.002 (Spearman's rho test), Figure 3C]. Representative case No.64 showed high PRL-1 and low E-cadherin expression levels; and vice versa was noted in case No.54. Taken together, these results suggest that PRL-1 regulates the expression of E-cadherin in HCC.

### PRL-1 inhibits the expression of E-cadherin in HCC by activating PI3K/AKT/GSK3β signaling pathway

PI3K/AKT signaling pathway modulates EMT [20], prompting us to examine this pathway in HCC cell lines using western blot assay. Exogenous overexpression of PRL-1 in Huh7 and SK-hep1 cell lines enhanced phosphorylation of AKT at Ser^474^,which resulted in increase phosphorylation of GSK3βand elevated levels of Snail expression and as previously noted, decreased E-cadherin expression (Figure 4A,B). In contradistinction, silencing of PRL-1 protein level using siRNA interference caused down-regulation of expression of p-AKT(Ser^474^), p-GSK3β(Ser^9^) and Snail transcriptional factor (Figure 4C).

To explore further whether PI3K/AKT signaling pathway is involved in PRL-1-mediated inhibition of E-cadherin expression, ZSTK474, a novel phosphatidylinositol 3-kinase (PI3K) inhibitor was utilized. Decrease of p-AKT (Ser^474^) after exposure of the cells to ZSKT474 reduced expression of p-GSK3β (Ser^9^) and Snail and restored E-cadherin expression (Figure 4D). Collectively, these observations support that PRL-1 can enhance the PI3K/AKT signaling pathway resulting in a reduction of E-cadherin expression in HCC cells.

### PI3K/AKT signals are potentially involved in PRL-1-enhanced HCC cell migration and invasion

To evaluate whether PI3K/AKT signals could mediate the invasive properties of PRL-1 in HCC, stably transfected Huh7-HA-PRL-1 cells were treated with PI3K inhibitor, ZSTK474 (10 uM) which abrogated the enhanced migration mediated by PRL-1 in HCC cells as measured by the wound-healing assay (Figure 4E) and the matrigel invasion assay (*p*<0.01, Figure 4F). These results provide evidence that an active PI3K/AKT signaling pathway can mediate the invasive activities of hepatoma cells that overexpress PRL-1.

### PRL-1 is potentially involved in metastatic process of primary liver cancer

**Figure5:**
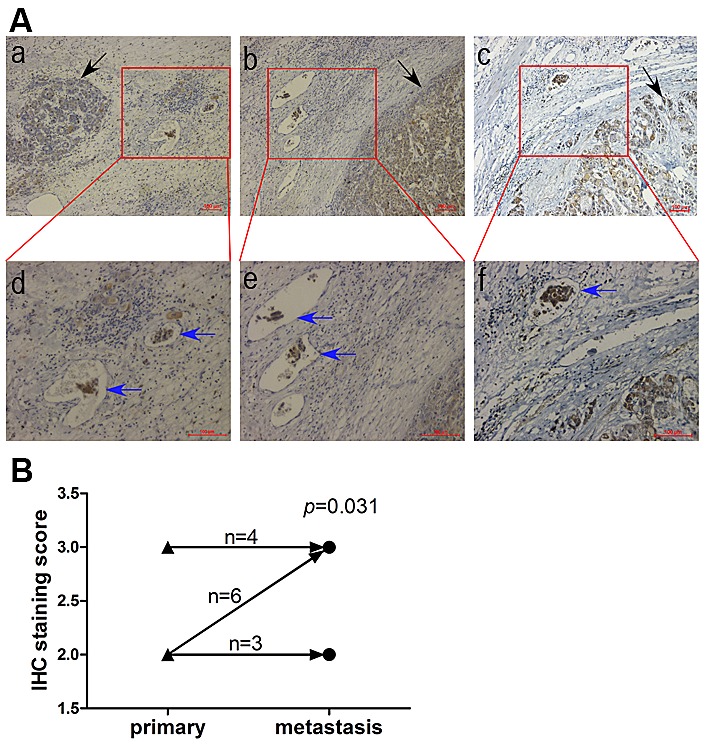
Comparison of PRL-1 expression in primary HCC and metastatic intravasular cancer thrombus by IHC A, Three illustrations showing the immunohistochemical staining in metastatic cancer thrombus (blue arrows) is stronger than that in primary lesion (black arrow). The upper panels, magnification (x100) and the lower panels, magnification (x200). B, A schematic diagram indicating that intravasular cancer embolus had higher PRL-1 expression than primary HCC (*p*=0.031). ▲, primary lesion. ●, metastatic intravasular cancer thrombus.

Tissue sections of 13 HCC cases with intravascular cancer thrombus were analyzed by IHC. A total of 10/13 (77%) of cases displayed high IHC staining of PRL-1 in the metastatic intravascular cancer thrombus. McNemar test showed that that IHC staining intensity of PRL-1 is stronger in metastatic lesion than in primary liver cancer (*p*=0.031). Three representative examples showed the strong IHC staining of PRL-1 in the intravascular cancer thrombus (Figure 5A). These findings are consistent with PRL-1 playing an important role in the metastatic process of primary HCC.

## DISCUSSION

A powerful approach to discover key genes playing causal roles in tumorigenesis is to identify genomic regions that undergo frequent alterations in human cancers [21]. High-density SNP-Chip arrays allow rapid detection of copy number changes in small regions of the cancer genome DNA, including amplifications, deletions, and acquired uniparental disomy (AUPD) [22,23]. In some cases, focal somatic copy number alterations (SCNAs) have led to the identification of cancer-causing genes [24-26]. Our high-density SNP-Chip arrays showed that seven of 60 HCC patients had PRL-1 copy number amplification. In addition, we observed by IHC that upregulation of expression of PRL-1 protein was a frequent event in HCC tissues. Together, the data implicated PRL-1 as a potential cancer-related gene in HCC.

Elevated PRL-1 levels were significantly associated with higher TNM stages and intravascular cancer thrombus in HCC, indicating a more aggressive tumor phenotype. Kaplan-Meier and Cox regression analysis suggested that increased expression of PRL-1 was an independent indicator of short post-surgical OS for patients with HCC. Functional analysis of PRL-1 in HCC cells demonstrated that overexpression of PRL-1 dramatically enhanced hepatoma cells migration and invasion.

Recurrence and metastasis are two related hallmarks of cancer [27]. Both are major causes of HCC-related mortility. Thus, identification the underlying molecular mechanisms are critical. EMT, is strongly hypothesized as a driver of cancer progression and metastasis [28]. This process results in the loss of epithelial properties including cell-cell adhesion and baso-apical polarity, and the gain of mesenchymal properties, which facilitates cell motility and invasion [29]. Among the markers of EMT, decrease of expression of E-cadherin has been recognized as a central molecule. Furthermore, loss of E-cadherin is correlated with tumor progression and metastasis in a variety of human cancers [30]. Our experimental data showed that exogenous expression of PRL-1 depressed E-cadherin expression level, while silencing of PRL-1 levels elevated E-cadherin expression at both mRNA and protein levels of HCC cells. Importantly, we found that expression levels of PRL-1 were significantly inversely correlated with expression levels of E-cadherin in HCC using tissue microarrays. These observations suggest that PRL-1 potentially accelerates liver cancer cells progression through inhibiting E-cadherin expression.

We dissected the molecular signaling pathway associated with ability of PRL-1 to repress expression of E-cadherin. Reintroduction of PRL-1 in Huh7 and SK-hep1 HCC cell lines caused phosphorylation of AKT at ser^474^, which subsequently inactivated GSK-3β at ser^9^ and elevated transcriptional factor Snail expression. Snail is known to be able to down-regulate transcription of E-cadherin. Consistent with this PRL-1 signaling pathway, the knockdown of PRL-1 expression in Huh7 cell line inhibited phosphorylation of AKT at ser^473^, activated GSK-3β at ser^9^, and decreased Snail expression, resulting in restoration of E-cadherin expression. In addition, treatment of PRL-1 stably-transfected HCC cells (Huh7) with PI3K inhibitor ZSTK474 remarkably repressed activities of phosphorylation of AKT at ser^474^, restored GSK-3βactivities, inhibited Snail expression, rescued the expression of E-cadherin, as well as repressed cells migration and invasion. In summary, elevated levels of PRL-1 in HCC cells activate the PI3K/AKT signaling pathway resulting in Snail repression E-cadherin transcription and enhanced migration and invasion of HCC cells. Furthermore, intravascular HCC thrombi had high PRL-1 expression, consistent with elevated PRL-1 acting as a pro-metastatic factor in HCC. Our data also suggest that PRL-1 may be an useful prognostic marker and/or an effective therapeutic target for HCC patients.

## MATERIALS AND METHODS

### DNA preparation and high-density SNP-Chip analysis

K-phenol-chloroform extraction method was used to extract DNA from HCC samples. Written informed consent for research use of all of these samples was obtained prior to surgery, according to a protocol approved by the institutional ethics committee. SNP-Chips for human 50k XbaI / 250k Nsp arrays were used for this study (SNP-Chip, Affymetrix, Santa Clara, CA, USA), as previous described [22,23]. The ArrayExpress accession number is E-MEXP-1330.

### Tissue specimens and tissue microarray

HCC and the adjacent normal liver tissue samples were obtained with informed consent under institutional review board-approved protocols. The samples were collected between June 2000 and September 2007 at the Sun Yat-sen Memorial Hospital, Sun Yat-Sen University (Guangzhou, China). The HCC cases selected were based on a clear pathological diagnosis, follow-up data; and the patients had not received previous local or systemic treatment. Tumour stage was defined according to the 2002 American Joint Committee on Cancer/International Union Against Cancer tumour-node-metastasis classification system. This study was approved by the institute research ethics committee of the Sun Yat-sen Memorial Hospital, Sun Yat-Sen University. Tissue microarray was constructed by He C. et al [31].

### Cell cultures and reagents

Three HCC cell lines (i.e., HepG2, Huh7 and SK-hep1) and 293T cell line were cultured in Dulbecco's Modification of Eagle's Medium (DMEM; Gibco, Carlsbad, CA, USA) supplemented with 10% fetal bovine serum (FBS; Gibco, Carlsbad, CA, USA). The stably transfected cell lines were cultured in DMEM supplemented with 10% FBS and 1 μg/mL puromycin (Sigama-Aldrich, St.louis, MO, USA). All cells were maintained at 37oC in a humidified incubator with 5% CO_2_.

### Immunohistochemistry (IHC)

The immunohistochemical study of PRL-1 and E-cadherin was performed using a standard two-step technique. Paraffin sections were dried for 20min at 68°C, dewaxed in xylene, rehydrated through graded alcohol, and immersed in 3% hydrogen peroxide for 15 min to block endogenous peroxidase activity. An antigen retrieval process was accomplished using hyperbaric heating (HH) repair with 10 mM citrate buffer (pH 6) for 3 min. The slides were incubated with 5% normal goat serum at room temperature for 30 min to reduce nonspecific reaction. Subsequently, the slides were incubated overnight at 4°C with either rabbit polyclonal antibody against PRL-1 (1:100; Abgent, USA) or rabbit monoclonal antibody against E-cadherin (1:100; Cell Signaling Technology, USA). After rinsing three times with 0.01 mol / L phosphate-buffered saline (PBS; pH = 7.4) for 10 min, the detection of the primary antibody was achieved by addition of a secondary antibody (Envision; Dako, Glostrup, Denmark) for 1 h at room temperature, and stained with DAB (3,3-diaminobenzidine) after washing in PBS again. Finally, the sections were counterstained with Mayer's hematoxylin, dehydrated, and mounted. PBC replaced primary antibody as a negative control.

### Construction of pMSCV-HA-PRL-1 plasmid

PCR fragments of HA-PRL-1 were inserted into BgiII and XhoI sites of the pMSCV-PIG vector provided by Dr. Mendel [32].

### Production of PRL-1 stable HCC cell lines

HepG2, Huh7, and SK-hep1 HCC cell lines were stably infected using pMSCV-PIG vector containing the human PRL-1 gene. HEK293T packaging cells were transfected with the appropriate retroviral construct using Lipofectamine 2000 (Invitrogen). Culture supernatants were collected 36 to 60 hours after transfection and filtered. Target cells were infected with the filtered viral supernatants in the presence of 6 μg/mL Polybrene for 48 hours, after which the medium was changed. Following infection, cells were selected with 4 μg/mL puromycin for 2 weeks, and the resistant population was used for cellular assays.

### Wound healing and invasion assays

Cell migration was assessed by measuring the movement of cells into a scraped, acellular area created by the tip of a 200 ul pipette, and the degree of “wound closure” was observed after 24h and photographed under a microscope. We measured the fraction of cell coverage compared to initiate gap and defined it as the migration rate. For invasion assays, 10^5^ cells were added to a Matrigel invasion chamber (BD Biosciences, New Jersey, USA) present in the insert of a 24-well culture plate. 10% FBS was added to the lower chamber as a chemo-attractant. After either 10 h (SK-hep1 cells) or 12 h (Huh7 cells), the non-invading cells were gently removed with a cotton swab. Invasive cells, located on the lower side of the chamber, were fixed with 4% paraformaldehyde (PFA) and stained with crystal violet, air dried and photographed. For colorimetric assays, the samples were treated with 600 ul 33% acetic acid, and the absorbance was measured at 570 nm with a spectrophotometer (Spectramax M5).

### RNA isolation and quantitative real-time PCR

Total RNA was extracted with TRIzol reagent (Invitrogen, Carlsbad, California, USA). cDNA was synthesized with the Prime Script RTase (Takara, Inc) according to the manufacturer's instructions. Real-time PCR for PRL-1, E-cadherin, and glyceraldehyde-3-phosphate dehydrogenase (GAPDH, an internal control) was performed on a LightCycler 480 system (Roche) using Premix Ex Taq (Takara, Inc) according to the manufacturer's instructions. Following primer pairs were used for each reaction: PRL-1, 5'-ACCAATGCGACCTTAAACAAA-3' (forward) and 5'-AATCTGGTTGGATGGTGGTG-3' (reverse); E-cadherin, 5'-CGTCCTGGGCAGAGTGAA-3' (forward) and 5'-GGCGTAGACCAAGAAATGGA-3' (reverse); GAPDH, 5'-AGCCACATCGCTCAGACAC-3' (forward) and 5'-GAATTTGCCATGGGTGGA-3' (reverse).

### RNA interference

Short interfering RNA specifically against PRL-1 [12,33] and the corresponding scrambled siRNA (GenePharma, Shanghai, China) were transfected into HCC cells in six-well plates using X-treme GENE siRNA Transfection Reagent (Roche, German) according to the manufacturer's instructions. Confirmation of silencing of target gene was measured by western blotting 48h post-transfection.

### Western blot assay

Cells were harvested and lysed in cell lysis buffer (50 mM Tris-HCl [pH 7.^4^], 250 mM NaCl, 0.1% NP-40, 5 mM EDTA, 2 ug/ml leupeptin, 2 ug/ml aprotinin, 4 mM Prefabloc SC, and protein inhibitor cocktail). Each 25 ug aliquot of denatured protein were separated by 10% SDS-PAGE, and then transferred onto a 0.22 um polyvinylidene difluoride membranes (Millipore). After completing protein transfer, the membrane was blocked in 5% (w/v) skimmed milk in TBST and incubated overnight at 4oC with the rabbit polyclonal antibody (IgG) against AKT, p-AKT (Ser^474^), GSK3β, p-GSK3β (Ser^9^), Snail, E-cadherin, PRL-1, and HA, respectively. The blots were detected by the secondary antibody, horseradish peroxidase (HRP)-linked polyclonal goat anti-rabbit IgG and visualized with SuperSignal West Dura (Thermo). GAPDH served as an internal loading control. Except that PRL-1 antibody was obtained from Abgent, all the other antibodies were purchased from Cell Signaling Technology. All antibodies were diluted to 1:1000.

### Immunofluorescence analysis

Cells were grown in glass-bottom dishes (Nest, Wuxi, China) to 50-70% confluence, washed three times with PBST, fixed in 4% PFA and processed for immunofluorescence staining. For IF staining, cells were first incubated with primary mouse anti-HA antibody (1:400 dilution) and rabbit anti-E-cadherin (1:200 dilution) overnight at 4°C. After thorough washing, cells underwent 1 hour incubation with fluorescence-conjugated secondary antibody (Pyld-conjugated goat anti-mouse, APC-conjugated goat anti-rabbit) at room temperature. Subsequently, cells were washed and stained with 0.5 ug/ml DAPI (Vector Laboratories, Burlingame, CA). Finally, cells were washed three times with PBST and photographed under a laser confocal microscope (Zeiss LSM710, German).

### Statistical analysis

Statistical analysis was performed using a SPSS software package (SPSS Standard version 13.0, SPSS Inc). Differences between variables were assessed by the Chi-square test. Survival analysis of patients with HCC was calculated by Kaplan-Meier analysis. A log rank test was used to compare different survival curves. Multivariate survival analysis was performed on all parameters that were found to be significant in univariate analysis using the Cox regression model. Data derived from cell-line experiments are presented as mean±SD (X±SD) and assessed by a two-tailed Student t test. *p* Values <0.05 were considered significant.
